# Alternative management of the left subclavian artery in thoracic endovascular aortic repair for aortic dissection: a single-center experience

**DOI:** 10.1186/s40001-015-0147-z

**Published:** 2015-05-31

**Authors:** Lei Zhang, Qingsheng Lu, Jian Zhou, Zaiping Jing, Zhiqing Zhao, Junmin Bao

**Affiliations:** Department of Vascular Surgery, Changhai Hospital, Second Military Medical University, Shanghai, China; Military Institute of Vascular Surgery, Changhai Hospital, Second Military Medical University, Shanghai, China

**Keywords:** Aortic dissection, Left subclavian artery, Thoracic endovascular aortic repair

## Abstract

**Background:**

Since the new 2009 guidelines for left subclavian artery (LSA) management using thoracic endovascular aortic repair (TEVAR), a few studies have been published about alternative LSA management. The objective of this study was to present the follow-up results of covered or revascularized LSA during TEVAR.

**Methods:**

From January 2010 to August 2012, 109 consecutive patients were treated with TEVAR at the Department of Vascular Surgery, Changhai Hospital, for aortic dissection extending near the LSA. After evaluating the bilateral vertebral arteries, fifty-two LSAs were covered and not revascularized (covered group), while 57 LSAs were preserved (revascularized group). Complications were stratified according to the time of occurrence after surgery.

**Results:**

Emergency operations were more common (17.3 vs. 3.5 %, *P* = 0.017) and operation time was shorter (96.9 ± 16.3 vs. 135.3 ± 38.4 min, *P* < 0.001) in the covered group. Pulselessness and intermittent claudication of the left arm occurred in most patients in the covered group (*P* < 0.001). Incidence of stroke and cold shoulder feeling were higher in the covered group compared with the revascularized group (*P* = 0.026 and <0.001, respectively). There were five aorta-related deaths in the covered group and one in the revascularized group. Eight endoleaks were observed in the revascularized group (*P* = 0.006).

**Conclusions:**

The results of this study suggest that due to occurrence of complications, LSA should be preserved or revascularized to reduce complications and to improve patients’ quality of life.

## Background

Aortic dissection is the disruption of the aortic media with bleeding within and along the aortic wall resulting in separation of aortic layers [1]. About two thirds of acute aortic dissections occur in men and in patients aged >60 years [2, 3]. Estimated annual incidence of aortic dissection is 2–6 per 100,000 individuals [4, 5]. Risk factors are hypertension, direct blunt trauma, pheochromocytoma, cocaine use, weight lifting, aorta coarctation, and some genetic syndromes [1, 2]. Aortic dissection may result in aortic rupture, aortic valve insufficiency, end-organ complications, and death [1, 2].

The advent of thoracic endovascular aortic repair (TEVAR) has altered the management algorithm for aortic dissections [6]. The increased use of TEVAR has been driven by advantages reported in older patients with greater comorbidities who have been judged unfit for direct open surgery and optimal medication regimens [7, 8].

An adequate length of proximal landing zone is a prerequisite for endovascular therapy [9]. Therefore, covering the left subclavian artery (LSA) with a thoracic stent graft to achieve an adequate landing zone is sometimes inevitable. However, there is a controversy in the literature regarding whether to simply cover the LSA or to revascularize it. Several studies have concluded that the risks associated with simply covering the LSA are low and that subclavian artery bypass could be performed in cases of obvious postoperative complications, such as the presence of left arm claudication or vertebrobasilar insufficiency [10–13]. Conversely, other studies identified an increased risk of neurologic complications, specifically strokes and spinal cord ischemia following LSA coverage [14–16].

In 2009, the Society for Vascular Surgery published the clinical practice guidelines for LSA management during TEVAR [17]. The guidelines proposed three recommendations to address the LSA. The first two guidelines are for elective TEVAR and suggest revascularization is the most suitable method. The third recommendation suggests that revascularization should be individualized and addressed on the basis of anatomy, urgency and availability of surgical expertise to patients who need very urgent TEVAR for life-threatening acute aortic syndromes where achievement of a proximal seal necessitates coverage of the LSA. However, revascularization can be performed after emergency TEVAR. Therefore, these guidelines do not answer the controversy, and more results are needed to assess this point.

On the basis of our previous experience in endovascular treatment and the branches of aortic arch [18–22], the present study aimed to assess the outcomes of patients who underwent TEVAR for aortic dissection and compare the outcomes of patients who had their LSA covered with those who had revascularized LSA.

## Methods

### Patients

This was a single-center retrospective study of patients with aortic dissection treated by TEVAR (*n* = 109) at the Vascular Surgery Department of Changhai Hospital, Shanghai, China, between January 2010 and August 2012. The study protocol was approved by the Ethics Committee of the hospital, and the need for individual consent was waived by the committee.

Aortic dissection diagnosis was confirmed by computed tomography angiography (CTA) [1, 2]. Indications for TEVAR were: (1) complicated aortic dissection, (2) symptomatic penetrating aortic ulcer, or (3) complete transection of the aortic wall and free bleeding [23].

The inclusion criteria were: (1) an aortic dissection diagnosis and (2) the patient underwent TEVAR. The exclusion criteria were: (1) congenital connective tissue diseases such as Marfan syndrome, (2) previous open surgery or endovascular therapy for aortic diseases, (3) intramural hematoma, or (4) asymptomatic penetrating ulcers.

### Study design

Patients were stratified into two groups according to LSA management. Fifty-two LSAs were covered and not revascularized (covered group). Fifty-seven LSAs were preserved (revascularized group) through bypass grafting [24], scallop or fenestration techniques [25], chimney techniques [19, 26, 27], or single-branch techniques [17].

### Outcome and follow-up

All patients were followed up with CTA of the aorta and the branches of the aortic arch at 6-month intervals for the first year and then once annually. The primary adverse events were stroke, paraplegia, and death. Follow-up was censored on December 2013.

### Statistical analysis

Statistical analysis was performed using SPSS 19.0 (IBM, Armonk, NY, USA). Categorical variables are presented as numbers and proportions, and were analyzed using chi-square or Fisher’s exact tests, as appropriate. Continuous variables are presented as means ± standard deviations or as median (range) and were analyzed using *t* tests or nonparametric tests, as appropriate. Event-free survival was analyzed using the Kaplan-Meier method and curves were compared using the log-rank test. Two-tailed *P* values <0.05 were considered significant.

## Results

### Patient characteristics

Patient characteristics are presented in Table 1. The mean age at onset was 56.2 ± 9.6 years, and majority of the patients were male (86.2 %). Ten patients were older than 70 years of age. The mean body mass index was 23.8 ± 3.2 kg/m^2^. Ninety-three patients had a history of hypertension, and 41 patients were smokers at the time of admission. Associated comorbidities were chronic obstructive pulmonary disease (*n* = 7), diabetes mellitus (*n* = 11), stroke (*n* = 4), myocardial infarction (*n* = 9), and angina (*n* = 5). The proximal entry tears were located in the proximal descending aorta in 62 patients, in the arch in 32 patients, and in the ascending aorta in 15 patients. Three patients underwent preoperative hemodialysis, and one patient had preexisting congestive heart failure in the revascularized group. Two patients had renal failure and seven patients had pneumonia. General anesthesia was administered to 10 patients in the covered group and 14 in the revascularized group. The number of emergency procedures was nine in the covered group and two in the revascularized group (*P* = 0.017). The time taken for the surgical procedure was 96.9 ± 16.3 min in the covered group and 135.3 ± 38.4 min in the revascularized group (*P* < 0.001).

**Table 1:** Patient characteristics

Variable	Overall	Covered group	Revascularized group	*P* value
*n* (%)	109	*n* = 52 (47.7)	*n* = 57 (52.3)	
Demographic/anthropometric factors				
Age, years	56.2 ± 9.6	55.5 ± 10.5	56.5 ± 9.3	0.636
Age >70, *n* (%)	10 (9.2)	6 (11.5)	4 (7.0)	0.514
Male, *n* (%)	94 (86.2)	43 (82.7)	51 (89.5)	0.305
Body mass index, kg/m^2^	23.8 ± 3.2	23.3 ± 2.0	24.0 ± 3.7	0.345
Medical history, *n* (%)				
Hypertension	93 (85.3)	43 (82.7)	50 (87.7)	0.459
Current smoker	41 (37.6)	24 (46.2)	17 (29.8)	0.079
Chronic obstructive pulmonary disease	7 (6.4)	5 (9.6)	2 (3.5)	0.255
Diabetes mellitus	11 (10.1)	6 (11.5)	5 (8.8)	0.632
Stroke	4 (3.7)	3 (5.8)	1 (1.8)	0.346
Myocardial infarction	9 (8.3)	4 (7.7)	5 (8.8)	>0.999
Angina	5 (4.6)	3 (5.8)	2 (3.5)	0.668
Preoperative hemodialysis	3 (2.8)	0	3 (5.3)	0.245
Congestive heart failure	1 (0.9)	0	1 (1.8)	>0.999
Acute preoperative conditions, *n* (%)				
Renal failure	2 (1.8)	0	2 (3.5)	0.496
Pneumonia	7 (6.4)	4 (7.7)	3 (5.3)	0.707
Procedural characteristics, *n* (%)				
Emergent procedure	11 (10.1)	9 (17.3)	2 (3.5)	0.017
Traumatic aortic dissection	1 (0.9)	1 (1.9)	0	0.477
General anesthesia	24 (22.0)	10 (19.2)	14 (24.6)	0.502
Operation time (min)	122.6 ± 37.4	96.9 ± 16.3	135.3 ± 38.4	<0.001

### Indications for alternative management of the LSA

The reasons for alternative management of the LSA are presented in Table 2. There were 38 patients (including five emergency procedures) with a dominant right vertebral artery confirmed by preoperative CTA. Eleven patients (including four emergency procedures) had equipotent bilateral vertebral artery. Three cases of LSA thrombosis were included in the covered group. The revascularized group was composed of 41 patients (including two emergency procedures) with a dominant left vertebral artery. Three patients who underwent preoperative hemodialysis had a functional arteriovenous shunt in the left arm. Other indications for revascularization of the LSA included an occluded right vertebral artery (*n* = 3), planned long-segment coverage of the descending thoracic aorta (*n* = 5), patent left internal mammary artery to coronary artery bypass graft (*n* = 2), and bilateral internal carotid artery stenosis (*n* = 3).

**Table 2:** Indications for alternative management of the left subclavian artery in both groups

Indications	*n* (%)
	*n* = 109
Covered group	
Dominant right vertebral artery (including 5 emergency procedures)	38 (34.9)
Bilateral vertebral artery equipotential (including 4 emergency procedures)	11 (10.1)
Preoperative thrombosis of the left subclavian artery	3 (2.7)
Revascularized group	
Dominant left vertebral artery (including 2 emergency procedures)	41 (37.6)
Occluded right vertebral artery	3 (2.7)
Planned long-segment coverage of the descending thoracic aorta	5 (4.7)
A functioning arteriovenous shunt in the left arm	3 (2.7)
Patent left internal mammary artery to coronary artery bypass graft	2 (1.9)
Bilateral internal carotid artery stenosis	3 (2.7)

### Selection of revascularization method

The methods selected to preserve the LSA in the revascularized group are shown in Table 3. Eleven patients underwent bypass grafting, six the scallop or fenestration techniques, 12 the chimney approach, and 28 received single-branched stent grafts.

**Table 3:** Method of revascularization for revascularized patients

Mode of revascularization	*n* (%)
	*n* = 57
Carotid-subclavian bypass or transposition	11 (19.3)
Scallop or fenestration	6 (10.5)
Chimney	12 (21.1)
Single-branched stent grafts	28 (49.1)

### Complications observed during follow-up periods

The median follow-up period was 34 months, ranging from 16 to 48 months. All of the preserved subclavian arteries remained patent, and all of the proximal entry tears were successfully occluded. No access site complications occurred. Complete thrombus formation in the false lumen of the aorta was demonstrated in all patients, and significant true lumen recovery and false lumen shrinkage were demonstrated in the aorta.

Complications during follow-up are presented in Table 4. Two strokes happened on the third and sixth day after the procedures, and five were observed during the mid- to long-term follow-up periods in the covered group (*P* = 0.026). Forty-six cases of pulselessness were observed; the patients had Doppler signals but no palpable pulses of the radial artery (*P* < 0.001), and twenty-four patients in the covered group suffered from intermittent claudication of the left arm when they performed physical activity (*P* < 0.001). Fifteen and two patients complained of a cold shoulder feeling in the covered group and revascularized group, respectively (*P* < 0.001). There was an aorta-related death in the covered group on the seventh day; the four other deaths in the group occurred on the second, fourth, fifth, and seventh month after the procedure. No abovementioned complication occurred in the revascularized group during short-term follow-up. However, some complications occurred during the mid- to long-term follow-up. Paraplegia was observed in four and two patients in the covered group vs. the revascularized group, respectively. A significant difference in endoleak occurrence was observed between the two groups (0 vs. 8, *P* = 0.006). The number of complications in the covered group was much higher compared with the revascularized group. In addition, patients in the covered group developed more complications in the third and sixth month after TEVAR, while the highest number of complications in the revascularized group occurred in the second month after TEVAR.

**Table 4:** All complications during follow-up

Complications	Covered group (*n* = 52)			Revascularized group (*n* = 57)			Sum I vs. sum II
	Early outcomes (<30 days)	Mid- to long-term outcomes (>30 days)	Sum I, *n* (%)	Early outcomes (<30 days)	Mid- to long-term outcomes (>30 days)	Sum II, *n* (%)	*P*
Stroke	2	5	7 (13.5)	0	1	1 (1.8)	0.026
Paraplegia	1	3	4 (7.7)	1	1	2 (3.5)	0.422
Pulselessness of left arm	3	43	46 (88.5)	0	0	0	<0.001
Endoleak	0	0	0	2	6	8 (14)	0.006
Intermittent claudication of left arm	4	20	24 (46.2)	0	0	0	<0.001
Cold shoulder feeling	2	13	15 (28.8)	0	2	2 (3.5)	<0.001
Aorta-related death	1	4	5 (9.6)	0	1	1 (1.8)	0.101

In the covered group, coils were used as an adjunctive technique in 16 patients; this method was used if a type II endoleak was caused by collateral reflux. The LSA orifice was then occluded by coils after puncturing the left brachial artery. We compared complication rates in the subgroup of patients treated with coils compared to those treated without coils but found there was no significant difference between them (Table 5).

**Table 5:** Major complications related with coils and location of entry tears during the follow-up period

Complications	Covered group (*n* = 52)		*P*	Location of entry tears in all patients (*n* = 109)			*P*
	With coils	Without coils		Ascending aorta	Aortic arch	Descending aorta	
	(*n* = 16)	(*n* = 36)		(*n* = 15)	(*n* = 32)	(*n* = 62)	
Stroke	3 (18.8)	4 (11.4)	0.662	2 (13.3)	4 (12.5)	2 (3.2)	0.166
Paraplegia	2 (12.5)	2 (5.6)	0.578	1 (6.7)	1 (3.1)	4 (6.5)	0.781
Aorta-related death	3 (18.8)	2 (5.6)	0.163	0	2 (6.3)	4 (6.5)	0.602

To evaluate whether the entry point location had an influence on the complication rate, we compared the number of complications for patients subgrouped according to the location of entry tears. The data are presented in Table 5 and show that there was no significant difference between them.

**Fig. 1:**
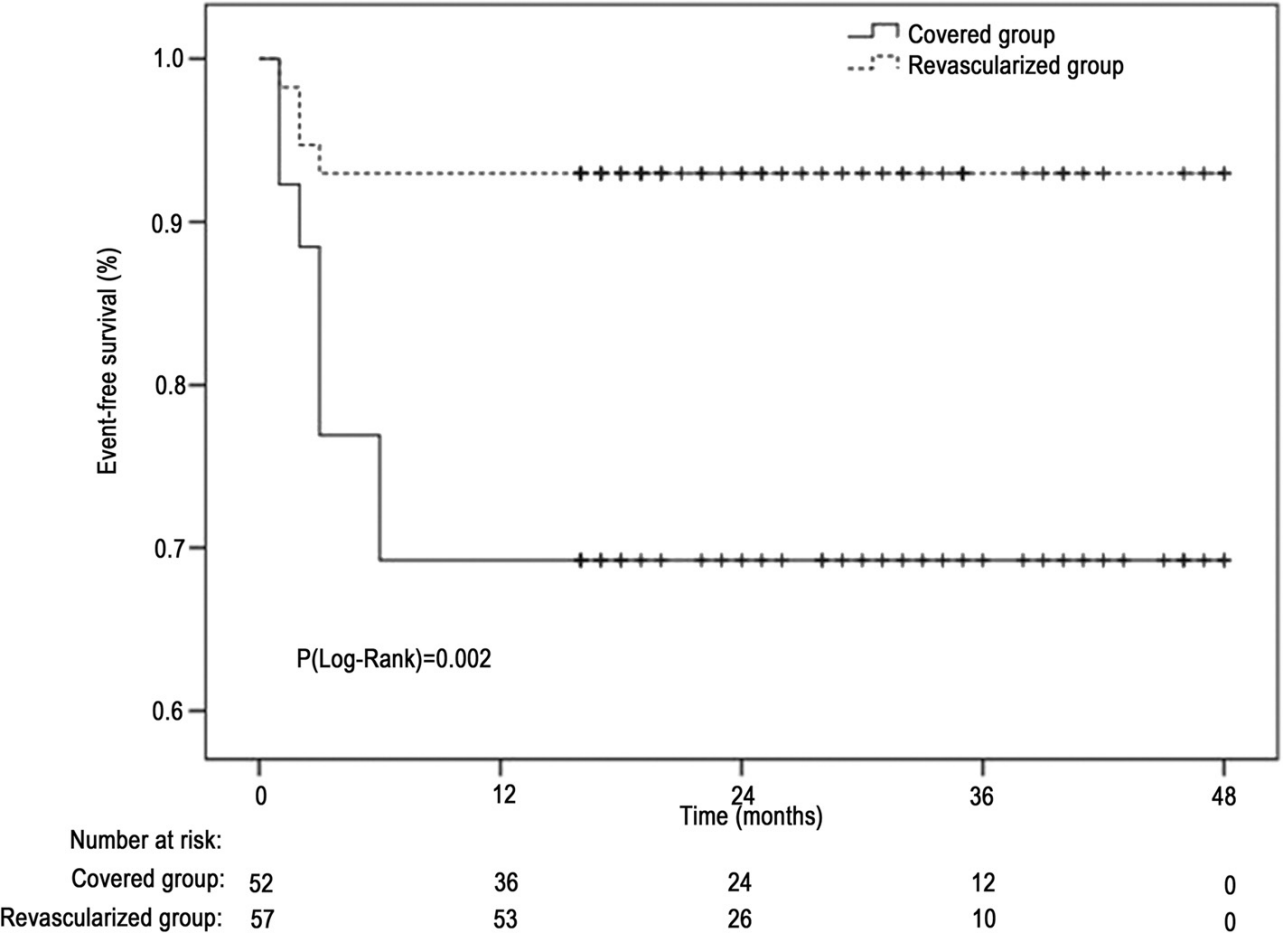
Kaplan-Meier curves of event-free survival in the covered and revascularized groups. Events were defined as stroke, paraplegia, or death

Figure 1 presents event-free survival. Compared with the covered group, the revascularized group had a better 4-year event-free survival (93.0 vs. 69.2 %, *P* = 0.002).

During follow-up, twelve patients in the covered group underwent revascularization of the LSA to improve their quality of life.

## Discussion

The objective of the present study was to present the follow-up results of covered or revascularized LSA during TEVAR. The results showed that emergency operations were more common and operation time was shorter in the covered group. Pulselessness and intermittent claudication of the left arm occurred in most patients in the covered group. The incidence of stroke and cold shoulder feeling were higher in the covered group compared with the revascularized group. There were five aorta-related deaths in the covered group and two in the revascularized group. Eight endoleaks were observed in the revascularized group.

To avoid complications induced by stent-graft migration, the seal zones of the stent grafts are required to be no less than 2 cm [9]. In nearly 40 % of all patients, this proximal landing zone involves covering the LSA [28]. However, the management of the LSA in the setting of intentional coverage during TEVAR remains controversial.

In 2009, the Society for Vascular Surgery developed the clinical practice guidelines for the management of the LSA with TEVAR and offered three main recommendations [17]. In the present study, different treatment strategies were used after evaluating the patients’ conditions and blood supply, based on the three recommendations from the guidelines. Results showed that most non-revascularized patients had left arm complications, such as pulselessness and intermittent claudication. In addition, over-stenting of the LSA without revascularization was associated with a relatively high incidence of stroke and a cold shoulder feeling compared with patients who underwent preoperative revascularization of the LSA.

The left vertebral artery originating from the LSA is a primary component of the vertebrobasilar artery, which divides into two posterior cerebral arteries and supplies two fifths of the blood to the brain [10]. More than 60 % of individuals have a dominant left vertebral artery, which has been used to justify routine preoperative LSA revascularization [15, 17, 29]. In addition, the guidelines underline that the LSA may be covered upon certain conditions [17]. Even in the absence of life-threatening symptoms, some benign symptoms may lower patients’ quality of life.

No other organ suffers more readily from an irreversible attack than the brain when its blood supply is insufficient. In those patients with a dominant right vertebral artery or equipotent bilateral vertebral arteries, covering the LSA would be devastating in the event of right vertebral artery stenosis or occlusion, such as by a thrombus resulting from atrial fibrillation. Indeed, the LSA is the primary artery to the left arm and a source of blood flow to the brain and spinal cord. Given the extensive circulation provided by the LSA, covering the LSA during TEVAR may not be inconsequential, which is associated with an increased risk of anterior and posterior stroke or spinal cord ischemia compared with patients whom this artery is not covered [8, 13, 15, 30]. A multicenter registry analysis concluded that the incidence of paraplegia due to spinal cord ischemia and stroke was higher in LSA-covered patients than in those who received prophylactic revascularization [31].

The guidelines suggest that the LSA must be revascularized in some situations [17] such as bilateral internal carotid artery disease, isolated left brain hemisphere, and an incomplete circle of Willis. In the present study, one patient was referred to our center urgently, eliminating the possibility of revascularizing the LSA. Unfortunately, the patient died on the seventh day after the emergency procedure due to an acute cerebral infarction. It is possible that this patient would have survived if postoperative revascularization had been performed.

The present study is not without limitations. First, it was a retrospective study performed in a small number of patients. In addition, a number of different approaches were used to revascularize the LSA, which could lead to bias. However, the present study analyzed the patients as patent/non-patent LSA. Further large multi-center studies are required to assess these points.

## Conclusions

Some complications were observed when covering the LSA during TEVAR. Therefore, the LSA should be preserved or revascularized if possible, whether preoperatively or postoperatively. In patients who are referred urgently, a postoperative revascularization should be executed when possible.

## Abbreviations

CTA: computed tomography angiography
LSA: left subclavian artery
TEVAR: thoracic endovascular aortic repair
